# Phosphoproteomic differences in major depressive disorder postmortem brains indicate effects on synaptic function

**DOI:** 10.1007/s00406-012-0301-3

**Published:** 2012-02-21

**Authors:** Daniel Martins-de-Souza, Paul C. Guest, Natacha Vanattou-Saifoudine, Hassan Rahmoune, Sabine Bahn

**Affiliations:** 1 Department of Chemical Engineering and Biotechnology, University of Cambridge, Tennis Court Road, Cambridge, Cambridgeshire, CB2 1QT UK; 2 Max Planck Institute of Psychiatry, Proteomics and Biomarkers, Munich, Germany; 3 Department of Neuroscience, Erasmus Medical Centre, Rotterdam, The Netherlands

**Keywords:** Major depression, Phosphoproteome, Proteome, Phosphorylation, Mass spectrometry, Postmortem

## Abstract

There is still a lack in the molecular comprehension of major depressive disorder (MDD) although this condition affects approximately 10% of the world population. Protein phosphorylation is a posttranslational modification that regulates approximately one-third of the human proteins involved in a range of cellular and biological processes such as cellular signaling. Whereas phosphoproteome studies have been carried out extensively in cancer research, few such investigations have been carried out in studies of psychiatric disorders. Here, we present a comparative phosphoproteome analysis of *postmortem* dorsolateral prefrontal cortex tissues from 24 MDD patients and 12 control donors. Tissue extracts were analyzed using liquid chromatography mass spectrometry in a data-independent manner (LC-MS^E^). Our analyses resulted in the identification of 5,195 phosphopeptides, corresponding to 802 non-redundant proteins. Ninety of these proteins showed differential levels of phosphorylation in tissues from MDD subjects compared to controls, being 20 differentially phosphorylated in at least 2 peptides. The majority of these phosphorylated proteins were associated with synaptic transmission and cellular architecture not only pointing out potential biomarker candidates but mainly shedding light to the comprehension of MDD pathobiology.

**Electronic supplementary material:**

The online version of this article (doi:10.1007/s00406-012-0301-3) contains supplementary material, which is available to authorized users.

## Introduction

Major depressive disorder (MDD) is characterized by feelings of low mood and self-esteem and by loss of interest or pleasure in activities [12]. The consequences of MDD include negative effects on work and social relationships and associated comorbidities such as substance abuse and anxiety, which results in an enormous financial burden on healthcare services. The combination of direct costs, mortality costs arising from depression-related suicides and costs associated with effects on the workplace were estimated to be over 80 billion dollars in the United States alone in the year 2000 [14]. MDD is now thought to be a leading cause of disability worldwide and has been hypothesized to be the most incident disease of the twenty-first century [12]. Despite the fact that a number of molecular and image-based studies have been performed, an understanding of the underlying pathophysiology is still lacking, and there are still no robust empirical means of increasing our ability to diagnose such conditions accurately.

The Human Proteome Organization (HUPO) emerged from the Human Genome Project as a means of understanding gene and protein functions that may lead to the understanding of diseases such as MDD and to the identification of diagnostic/prognostic biomarkers [44]. The human genome is now known to contain approximately 35,000 genes [26, 58], although the number of proteins is anticipated to be at least one order of the magnitude greater. One reason for this high number is due to the fact that proteins can undergo posttranslational modifications such as phosphorylation [48], which can give rise to multiple forms of the same gene product.

Recent advances have been made in the development and application of large-scale molecular profiling techniques such as transcriptomics [43] and proteomics [4, 38, 39] in studies of biological tissues from MDD patients. However, there have only been a few studies on differential patterns of protein phosphorylation in MDD [38]. Phosphorylation controls a diverse range of cellular processes such as cell signaling via switching mechanism of the kinase-mediated addition of a high energy phosphate group to a serine, threonine or tyrosine residue on a protein. The large-scale analyses and quantification of phosphoproteins and/or phosphopeptides using mass spectrometry are known as phosphoproteomics and have been employed mostly in studies of cancer [2, 10] and neurodegenerative disorders studies [9, 15]. For instance, the hyperphosphorylation of TAU is one of the central mediators of Alzheimer’s disease (AD) pathogenesis. Studies of TAU phosphorylation have proven to be an effective example of how to discover molecular mechanisms and signaling pathways involved in pathogenic processes in the brain. Phosphoproteomics offer us the possibility of investigating in a large-scale manner the functional role of proteins, which is a subject mostly neglected in large-scale proteome studies. Investigation of changes in the phosphorylation states of proteins, which are independent of changes in their total expression, can provide insights about molecular signaling and mechanisms such as neuroplasticity and synaptic transmission.

Here, we have carried out a differential phosphoproteomic analysis of postmortem dorsolateral prefrontal cortex (DLPFC) tissue from MDD patients (*n* = 24) compared to matched controls (*n* = 12) using liquid chromatography mass spectrometry in a data-independent mode (LC-MS^E^). Our interest in the DLPFC arises from the important role that this brain structure plays in MDD pathogenesis [24]. It was of particular importance to determine whether differential phosphorylation is involved in the pathogenesis of MDD and whether such molecules might be used as potential biomarker candidates [32] as a means of developing novel molecular biomarker tests to improve diagnosis and for use as surrogate biomarkers in drug discovery studies.

## Methods and materials

### Brain tissue samples

*Postmortem* DLPFC tissues (Brodmann area 9) from 24 MDD patients and 12 matched control subjects were obtained from the Stanley Medical Research Institute brain collection (Bethesda, MD, USA) (Table 1 and Supplementary Material 1). Consent was obtained by questionnaire-based telephone interview and signed by the interviewer and a witness. The Institutional Review Board at the Uniformed Services University of Health Sciences determined that the procedure was exempt from federal and state regulations governing human research, since specimens were obtained from cadavers and anonymized with respect to personal information.

### Sample preparation

Brain tissue samples (20 mg) were homogenized individually in 100 μL of 7 M urea, 2 M thiourea, 4% CHAPS, 2% ASB-14 and 70 mM DTT [40] using the Sample Grinding Kit (GE Healthcare; Little Chalfont, Bucks, UK). Samples were centrifuged for 10 min at 16,000×*g*. The supernatants were collected and protein concentrations determined using the Bradford dye-binding assay (Sigma; Poole, Dorsett, UK).

### Shotgun LC-MS^E^ proteomics workflow

The following workflow was established previously [37]. Protein samples (15 μg) were subjected to sodium docecylsulfate polyacrylamide gel electrophoresis (SDS-PAGE) for pre-fractionation to enhance phosphoproteome coverage. Protein bands were visualized using Coomassie blue staining, and each lane containing stained protein bands was sliced to produce 3 horizontal sections. Gel sections were subjected to trypsin digestion in situ and resulting peptide mixtures were lyophilized. The peptides were suspended in 0.1% formic acid and injected (0.5 μg) in duplicate into a nano Ultra Performance Liquid Chromatography instrument containing a BEH-130 C18 column (75 μm × 200 mm) at a flow rate of 0.3 μL/min connected online to a Q-TOF Premier Mass Spectrometer (Waters Corporation; Manchester, UK). Eluted peptides were measured in MS^E^ mode (data-independent analysis) using the ion accounting algorithm [28] for data processing. Analysis of the resulting chromatograms/mass spectra and database searching were performed using the ProteinLynx Global Server (PLGS) v.2.4 (Waters Corp.). Quantitative and statistical analyses were performed using the Rosetta Elucidator^©^ system v.3.3.0.1.SP3.19 (Rosetta Inpharmatics; Seattle, WA, USA).

### Statistical analyses

Wilcoxon signed-rank test was used to determine significant differences between the groups under comparison (*p* < 0.05) in case data are not normally distributed. False discovery rate (FDR) was calculated according to Benjamini and Hochberg [5]. No adjustments were made for multiple comparisons as previously supported [53]. This approach is to avoid the exclusion of possible true positives since proteomic data are not necessarily random but can be physiologically interdependent observations, even though a Q-value threshold of approximately 0.4 and a fold change cut off of 10% have been established.

Considering that MDD and controls groups are matched for demographic variables (Table 1), results here are unlike to have suffered influence of gender, age, alcohol abuse, smoking, postmortem interval and refrigeration interval. By using principal component analysis (PCA), we could observe that potential interferences of medication are also unlikely (Supplementary Material 1).

**Table 1:** Demographic information for the samples used in the study (mean ± SD)

	Control	MDD	Significance *t* test (*p*)
Sample size	12	24	
Age	47 ± 12	42 ± 11	0.38
Postmortem interval	25.3 ± 10.6	29.7 ± 12.4	0.44
Refrigerator interval	7.4 ± 5.4	7.9 ± 6.3	0.80
Brain pH	6.6 ± 0.2	6.7 ± 0.2	0.44

			Fisher’s exact
Gender (male/female)	8/4	13/11	0.57

### Phosphoproteome analyses

Potential phosphorylated molecules were identified automatically by PLGS based on the experimentally determined loss of a 80 Da PO3− ion from peptides containing one or more phosphorylation consensus sequences featuring serine, threonine or tyrosine residues. Quantification was performed using the Elucidator^©^ system.

### Western blot validation

Brain tissue samples were prepared as described above. Samples were arranged in randomized order such that each of the two diagnostic groups (Controls vs. MDD) was represented on each gel. For each sample, 40 μg of total protein was electrophoresed using pre-cast Novex 10–20% Tricine polyacrylamide gels (Invitrogen; Paisley, UK) at 150 V for about 60 min, followed by semidry electrophoretic transfer to Immobilon-FL polyvinyldiphenyl fluoride (PVDF) membranes (Millipore; Watford, UK). The membranes were incubated in a 1:1 mixture of Odyssey blocking buffer (Li-COR Biosciences; Cambridge, UK). Membranes were then incubated overnight at 4°C with anti-Dynamin 1 (phospho S774) antibody (ab55324) at 1/500 dilutions (Abcam; Cambridge, UK). The membranes were washed in Tris-buffered saline (TBS) containing 0.1% Tween-20 for 1 h at room temperature (4 × 20 min) and then incubated for 1 h at room temperature with the appropriate IR-dye-conjugated secondary antibodies (1:7,500 for secondary rabbit antibody Li-COR Biosciences) in blocking buffer. Immunoreactive protein bands were visualized using the Odyssey Infra-red imaging system (Li-COR Biosciences) and the integrated intensities (II) of the bands measured. Values which lay outside the mean by more than twice the standard deviation were excluded from the analysis.

### Biological classification

Differentially phosphorylated proteins in MDD DLPFC were classified according to their biological pathways and subcellular localization using the Human Protein Reference Database (http://www.hprd.org). For interpreting functional significance of differentially phosphorylated proteins, the associated SwissProt accession identification codes for each phosphoprotein were uploaded into the Ingenuity Pathways Knowledge Base (IPKB) (http://www.ingenuity.com), and these were analyzed to identify potential interactions between these proteins and other proteins in the IPKB and for determining the most significant biological, disease and canonical pathways associated with these proteins (significance determine using Fisher’s exact test).

## Results

Using our shotgun LC-MS^E^ approach, we could identify 5,195 phosphopeptides in all the 36 analyzed samples corresponding to 802 distinct proteins. Comparing MDD patient samples to controls, significant differences in phosphorylation levels (*p* < 0.05—Wilcoxon signed-rank test) were observed for 116 phosphopeptides, corresponding to 90 distinct proteins (Table 2). Ten of these proteins (11.1%—Table 2 in black) were found with differences in protein expression, which impairs the confirmation of their differential phosphorylation. Fifty-three proteins (58.9%) presented phosphorylation differences in one phosphorylation site in a single peptide, while 17 proteins (18.9%) presented phosphorylation differences in at least 2 phosphorylation sites in a single peptide. More consistently, 20 proteins (22.2%) were differentially phosphorylated in more than 1 peptide (Table 2 in gray). For 3 of them—alpha-crystallin B chain (CRYAB), 60 kDa heat shock protein, mitochondrial (HSPD1) and myelin basic protein (MBP)—phosphopeptides presented phosphorylation differences in opposite directions. The presented results are not likely to have suffered neither interference of demographics (Table 1) nor medication according to a PCA which did not show clustering of samples (Supplementary material 1).

**Table 2:**
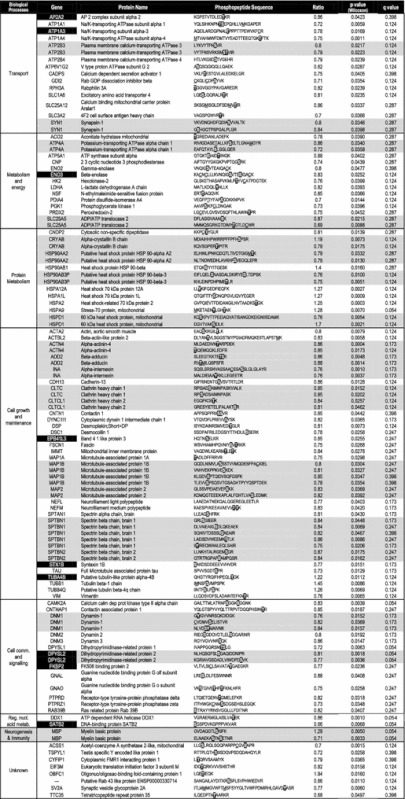
Differentially phosphorylated peptides identified and their correspondent proteins

In black, proteins found with differences in expression (42). In gray, proteins found differentially phosphorylated in more than 1 peptide

**Fig. 1:**
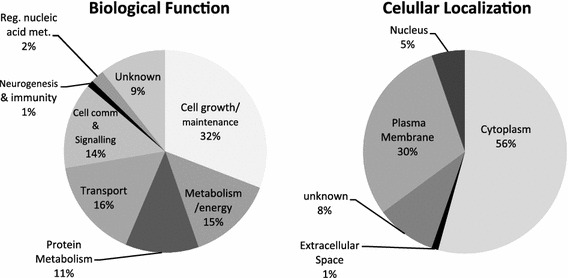
**a** Biological function and **b** cellular localization of the differentially phosphorylated proteins in MDD brains

**Fig. 2:**
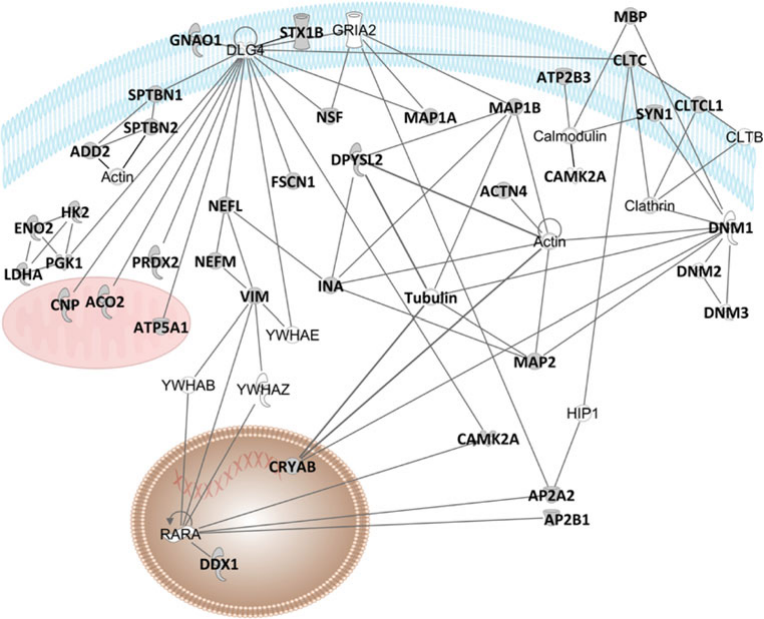
Network of proteins interactions among the differentially phosphorylated proteins according to systems biology analyses by ingenuity pathways knowledge base

**Fig. 3:**
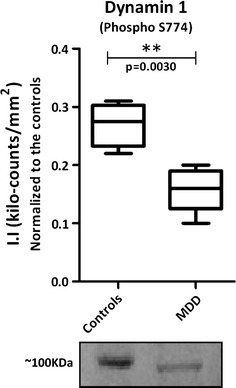
Validation of Dynamin 1 as a candidate of differentially phosphorylated protein in the DLPFC from MDD patients and controls. *p* values were obtained by Student’s *t-*test statistical analysis

These phosphoproteins were assigned according to their biological processes in order to comprehend the biochemical pathways associated with the differential phosphorylation signaling (Fig. 1). Seven different biological processes were represented, being “cell growth and maintenance” the most frequent class (Fig. 1a). Differentially phosphorylated proteins were also classified according to their cellular localization (Fig. 1b). Although most of them are cytoplasmic (56%), there was a significant coverage of membrane proteins (30%), which are important targets not only for protein signaling, but for potential drug targets. Phosphoproteins were also submitted to a systems biology analyses in IPKB as to be discussed ahead (Fig. 2). Considering the large-scale nature of our analyses, we performed a Western blot validation of phosphorylated Dynamin 1, confirming the LC-MS^E^ findings (Fig. 3).

## Discussion

Despite their obvious importance, there is still a lack about phosphoproteomic studies in psychiatric disorders [38]. It is estimated that protein phosphorylation regulates approximately one-third of the human proteins in a wide range of cellular processes [59]. Moreover, phosphoproteomic analyses could provide information about phosphorylation status, increasing the understanding about the functional aspects of the MDD. Even not using any special preparation for this purpose, we evaluate the phosphoproteomic differences in MDD compared to controls. We focused our discussion and our illustrations in the 20 proteins that showed differences in phosphorylation in at least 2 peptides, since those provide more confidence regarding differential phosphorylation. Nevertheless, the remaining 70 proteins are also important protein targets to be considered, proved by the fact that they fit in a biological and molecular context as further presented.

**Fig. 4:**
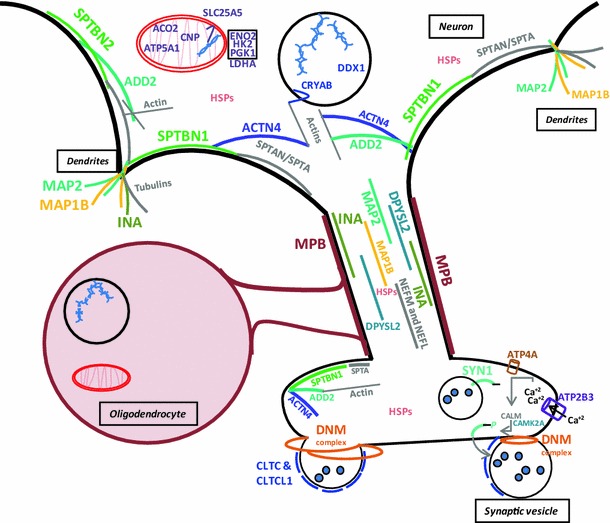
Integrated view of the role players of the synaptic dysfunction in MDD brains. Colored proteins were found differentially phosphorylated between MDD patients versus controls

The identification of differential phosphorylation in subunits of clathrin (CLTC and CLTCL1), spectrin (SPTBN1 and SPTBN2) and synapsin (SYN1) as well as the identification and validation of dynamin (DNM1) (Fig. 3) reinforces the impairment of synaptic transmission in MDD [16]. As represented in Fig. 4, our phosphoproteomic findings suggest a generalized dysregulation of the cytoskeleton signaling, which may compromise cell morphology and synaptic transmission. SPTBN1 and SPTBN2 together with other spectrin subunits like spectrin alpha chain (SPTAN1) are responsible for connecting plasma membrane to the actin cytoskeleton, playing roles in cell morphology, organelles organization and transmembrane proteins arrangement [6]. Alpha-actinin-4 (ACTN4) also belongs to the spectrin superfamily playing complementary roles to SPTBN1 and SPTBN2 and has been implicated in central nervous system (CNS) disorders such as schizophrenia and epilepsy [46]. Beta-adducin (ADD2) also plays structural roles in binding and regulating actin and spectrin filaments and contains phosphorylation sites for protein kinase C and calmodulin binding site [7]. Interestingly, Add2 KO mice presented differences in expression and phosphorylation levels of alpha- and gamma-adducin as well as impairments in synaptic plasticity, motor coordination, behavioral and learning deficits [51]. In addition, mRNA of sodium potassium transporting subunit alpha-3 (ATP1A3), calcium calmodulin dependent protein kinase type II alpha chain (CAMK2A) and vimentin (VIM) were found differentially expressed in MDD DLPFC [57].

Microtubule-associated proteins (MAPs) such as MAP1B and MAP2, which we found differentially phosphorylated in MDD DLPFC, bind to tubulin subunits for regulating microtubules assembly and stability. These are necessary for several cellular processes in brain tissue such as axonogenesis and dendritogenesis during neurodevelopment and function [1] as supported by studies in MAP1B null mice [8]. Moreover, MAP2 acts stabilizing and shaping dendrites during neuron development [13]. Interestingly, we found MAP1B less phosphorylated in MDD brain, in line with previous functional studies which suggested that a dephosphorylated state of MAP1B may trigger cytoskeletal alterations that will impair long-term potentiation leading ultimately to impaired synaptic plasticity [62]. In addition, MAP1B null adult mice presented alterations in myelin sheath diameter as well as conductance velocity of peripheral axons in the corpus callosum, which is in line with the differential phosphorylation we found for MBP in MDD DLPFC [8]. Stress-unsusceptible rats from a chronic social defeat stress model presented lower levels of Map1b mRNA in the frontal cortex and hippocampus [20]. Although we did not find significant differences in proteins expression when comparing MDD and control subjects, the reduced gene expression in the social defeat model and differential protein phosphorylation in brain tissue reinforce the role of MAP1B in the impairment of the synaptic transmission in MDD. Moreover, the inhibition of GSK-3beta by lithium leads to the loss of phosphorylated MAP-1B and thus to axonal remodeling [30], suggesting MAP1B as a potential drug target.

MAP1B is also involved in growth cone and axonal activity [45] so as alpha-internexin (INA) and dihydropyrimidinase-related protein 2 (DPYSL2). INA, which is also present in dendrites, is one of the responsible proteins to maintain neuronal caliber and is involved in neuronal morphogenesis together with neurofilaments L, M (NEFL and NEFM) [11], which we also found differentially phosphorylated. DPYSL2, which acts in the regulation of axon guidance, vesicle trafficking and synaptic function, has been shown to bind and be modulated by antidepressants and neuroactive molecules [19]. INA and DPYSL2 have been found differentially expressed in schizophrenia brains [34, 35, 41]. While their phosphorylation status has not been mentioned on schizophrenia, differences in expression of these proteins have not been related to MDD so far.

Synapsin-1 (SYN1) is a neuronal membrane phosphoprotein that anchors to synaptic vesicles which is involved in axogenesis, synaptogenesis and modulation of neurotransmitter release [56] so as *N*-ethylmaleimide-sensitive fusion protein (NSF), which is a key synaptic component especially in synaptic transmission SNARE-mediated [60]. SYN1 has been implicated in psychiatric disorders and could be a target for their treatment [61]. Synaptic vesicles reserved in the axon are converted to ready-to-release vesicles depending on SYN1 phosphorylation. When pre-synaptic membranes depolarize, there is a calcium influx into the axonal nerve, which can involve plasma membrane calcium-transporting ATPase 3 (ATP2B3) that we also found to be differentially phosphorylated. Calcium ions bind to calmodulin, and the complex calcium/calmodulin activates protein kinases that phosphorylate SYN1. In turn, SYN1 dissociates from the vesicle membrane, leading it for release [25, 54] (Fig. 4). Interestingly, SYN1 interacts with amphiphysin (AMPH) [47], which we found to be differentially expressed in MDD brains [36].

When ready-to-release synaptic vesicles reach the axonal membrane, they are mechanically involved by dynamins like DNM1, DNM2 and DNM3, which forms a spiral around the vesicle forcing their rupture at the expense of GTP hydrolysis [18] (Fig. 4). Interestingly, differences in the phsophorylation levels of clathrin heavy chain 1 and 2 (CLTC and CLTCL1) were also observed in MDD DLPFC. Clathrin complex performs a pivotal role in shaping synaptic vesicles and can be recycled after vesicle cycles also playing roles in axon guidance and organization of cellular membrane [29]. The roles of DNM1 and CLTC as well as the pivotal role of NSF can be observed in http://stke.sciencemag.org/content/vol2004/issue264/images/data/re19/DC2/slowtrack2.swf. Differences in the phosphorylation levels of key proteins such as DNM1 and CLTC can impair significantly synaptic transmission.

Although synaptic transmission is a known mechanism in MDD, revealing exactly which proteins, peptides and amino acids are differentially phosphorylated can lead to potential targets for therapeutic studies, paving way to the discovery of new drugs.

MBP, which is the major structural component of the myelin sheets in the CNS, and alpha-crystallin B (CRYAB) were identified with differential phosphorylation by two peptides, being one more phosphorylated and the other less phosphorylated. MBP gene and protein expression have been found altered consistently in schizophrenia [1, 31, 33, 42] and multiple sclerosis [55]. In addition, the differential expression of MBP in the entorhinal cortex in schizophrenia patients correlates to migrational disturbances of pre-alpha cell clusters leading to deficits in axonal myelination and disturbed connectivity during neurodevelopment [49]. CRYAB was also previously found differentially expressed in schizophrenia [41]. Therefore, the differential phosphorylation of MBP and CRYAB protein modification cannot point out a process specific for MDD, but can indicate trait processes of neuropsychiatric disorders. Considering the structural role of these proteins added to the cytoskeleton impairment described above, we here present the possible players of the impaired cytoarchitecture of MDD brain tissue. Recently, there has been found a correlation between the phosphorylation of CRYAB with apoptosis in breast cancer cells [27]. In addition, CRYAB acts as molecular chaperone [50], and the differential phosphorylation of other 3 heat shock proteins here found—putative heat shock protein HSP 90-beta-3 (HSP90AB3P), putative heat shock protein HSP 90-alpha A2 (HSP90AA2) and 60 kDa heat shock protein, mitochondrial (HSPD1)—may suggest impaired cellular environment. Interestingly, CRYAB has an autokinase activity, and posttranslational modifications decrease their role as chaperone [21].

Energy metabolism pathways have been associated intimately with MDD [3, 22, 52]. In line with these results, we observed recently that several oxidative phosphorylation enzymes were differentially expressed in MDD [36]. We found differences in the phosphorylation levels of metabolic enzymes such as aconitate hydratase (ACO2), enolase (ENO2 and ENO3), hexokinase-2 (HK2) and L-lactate dehydrogenase A (LDHA).

As for any postmortem brain tissue research, some potential limitations of our study have to be addressed. Factors such as age, gender, postmortem interval and others may confound global proteomic analyses especially in postmortem studies [23]. However, these factors are unlikely to have had significant effects on our analyses, considering that the compared groups are matched for demographic variables and these have not shown significant differences (Table 1). In addition, no segregation of subjects has been observed using principle component analyses for medication effects (Supplementary material 1). The limited sample sizes can also be a drawback in postmortem brain tissue studies, suggesting, therefore, replication in an independent sample cohort. In addition, it is suggested validation in a larger number of samples for overcoming errors from multiple testing. Even with all potential drawbacks, we believe that the analysis of postmortem tissue from patients while studying brain disorders is indispensable as reported recently [17]. These studies have generated insights for psychiatric studies that have been useful in basic and applied research.

Interestingly, the differences in phosphorylation that we observed in MDD DLPFC brain tissue can be connected in a net of interactions and roles as described in Figs. 2 and 4. The advantage of a hypothesis-free analysis as we performed is to give the opportunity to reveal the real players of the studied disorder. Moreover, considering the signaling role of phosphorylation, which is indispensable for cellular structure, neuroplasticity and communication, the protein candidates presented here be considered therapeutic targets to be further explored in drug response and drug discovery studies.

## Electronic supplementary material

Below is the link to the electronic supplementary material.

Supplementary material 1 (PDF 426 kb)
