# Supplementary material for: Phosphoproteomic differences in major depressive disorder postmortem brains indicate effects on synaptic function
Source: Eur Arch Psychiatry Clin Neurosci. 2012 Feb 21;262(8):657–66. doi: 10.1007/s00406-012-0301-3 (PMC3491199; doi:10.1007/s00406-012-0301-3)

Supplemental Material 1: Patients' demographics. TOD= time of death;

Diagnostic	Age	Gender	Cause Of Death	Post mortem interval	Brain pH	Age On Onset	Alcohol Use	Drug Use	Smoking At TOD	Mood Stabilizer (being taken at TOD)	Lifetime Antipsychotics	Antidepressant
DEPRESSIVE DISORDER	53	Male	CARDIAC	21	6.64	45	Insufficient info	Moderate in the past	Unknown			
DEPRESSIVE DISORDER	45	Male	SUIC: HANGING	29	6.75	30	Moderate in the past	Little or none	No			TRAZODONE
DEPRESSIVE DISORDER	56	Female	BURNS	15	6.59	25	Social	Heavy in the past	No			Insufficient info
DEPRESSIVE DISORDER	24	Male	SUIC: OVERDOSE	21	6.61	14	Insufficient info	Insufficient info	Yes	GABAPENTIN	Insufficient info	VENLAFAXINE
DEPRESSIVE DISORDER	45	Female	SUIC: OVERDOSE	13	6.58	37	Heavy in the past	Moderate in the present	Unknown	GABAPENTIN	100 (QUETIAPINE)	BUPROPION
DEPRESSIVE DISORDER	33	Male	SUIC: HANGING	25	6.86	17	Social	Moderate in the past	Unknown	VALPROATE		
DEPRESSIVE DISORDER	56	Male	SUIC: OVERDOSE	38	6.59	51	Moderate in the present	Little or none	No		1000 (RISPERIDONE)	VENLAFAXINE
DEPRESSIVE DISORDER	32	Female	SUIC: HANGING	19	6.8	32	Little or none	Little or none	Unknown			TRAZODONE
DEPRESSIVE DISORDER	44	Male	CARDIAC	24	6.52	31	Social	Little or none	Unknown	VALPROATE	3000 (CHLORPROMAZINE & OLANZAPINE)	BUPROPION
DEPRESSIVE DISORDER	34	Male	SUIC: FALLING	24	6.79	14	Heavy in the present	Little or none	Unknown			FLUOXETINE
DEPRESSIVE DISORDER	45	Female	CARDIAC	29	6.9	27	Little or none	Little or none	Unknown			SERTRALINE
DEPRESSIVE DISORDER	47	Female	SUIC: GUNSHOT	25	6.88	44	Little or none	Little or none	No			BUPROPION
DEPRESSIVE DISORDER	28	Male	SUIC: HANGING	26	6.7	20	Little or none	Little or none	Yes		3000 (RISPERIDONE)	FLUOXETINE
DEPRESSIVE DISORDER	36	Female	PULM EMBOL	32	6.74	25	Social	Insufficient info	Yes		2500 (RISPERIDONE)	TRAZODONE
DEPRESSIVE DISORDER	32	Female	SUIC: HANGING	19	6.7	22	Heavy in the present	Moderate in the present	Unknown		100	
DEPRESSIVE DISORDER	48	Female	SUIC: OVERDOSE	24	6.36	39	Social	Little or none	Yes		6500 (THIOTHIXENE)	DESIPRAMINE
DEPRESSIVE DISORDER	63	Male	SUIC: HANGING	31	6.6	59	Heavy in the past	Little or none	No		4000	NEFAZODONE
DEPRESSIVE DISORDER	40	Female	SUIC: HANGING	49	6.72	25	Insufficient info	Heavy in the past	Unknown		1000	VENLAFAXINE
DEPRESSIVE DISORDER	51	Female	UNKNOWN	36	6.3	30	Social	Little or none	Unknown	LITHIUM	700	TRANYLCYPROMINE
DEPRESSIVE DISORDER	35	Male	SUIC: HANGING	19	6.6	32	Little or none	Little or none	Unknown		2000	
DEPRESSIVE DISORDER	28	Female	SUIC: OVERDOSE	40	6.68	13	Moderate in the past	Little or none	Yes		Insufficient info	
DEPRESSIVE DISORDER	35	Male	SUIC: GUNSHOT	36	6.6	18	Heavy in the present	Moderate in the present	Unknown			
DEPRESSIVE DISORDER	40	Male	OVERDOSE	52	6.48	32	Heavy in the present	Heavy in the past	No	LITHIUM & VALPROATE	3000 (QUETIAPINE)	
DEPRESSIVE DISORDER	62	Male	SUIC: STABBED	65	6.57	35	Heavy in the present	Little or none	Unknown			NEFAZODONE
CONTROL	34	Male	CAR ACCIDENT	9	6.56	N/A	Little or none	Little or none	Yes	0	0	0
CONTROL	50	Male	CARDIAC	11	6.5	N/A	Social	Social	Unknown	0	0	0
CONTROL	24	Male	CAR ACCIDENT	17	6.6	N/A	Heavy in the past	Heavy in the past	Unknown	0	0	0
CONTROL	50	Female	CARDIAC	35	6.31	N/A	Little or none	Little or none	Yes	0	0	0
CONTROL	39	Female	CARDIAC	24	6.88	N/A	Little or none	Little or none	Unknown	0	0	0
CONTROL	56	Female	CARDIAC	29	6.78	N/A	Little or none	Little or none	No	0	0	0
CONTROL	48	Male	CARDIAC	12	6.51	N/A	Heavy in the past	Social	No	0	0	0
CONTROL	44	Male	ALCOHOL POISONING	27	6.82	N/A	Heavy in the present	Moderate in the present	Unknown	0	0	0
CONTROL	35	Male	CAR ACCIDENT	31	6.59	N/A	Moderate in the present	Little or none	Yes	0	0	0
CONTROL	63	Male	CARDIAC	40	6.91	N/A	Heavy in the past	Little or none	No	0	0	0
CONTROL	56	Female	OBESITY-RELATED	31	6.66	N/A	Heavy in the past	Little or none	Yes	0	0	0
CONTROL	63	Male	CARDIAC	37	6.5	N/A	Little or none	Little or none	Unknown	0	0	0

Supplemental Material 1: PCA analysis according to mood stabilizer medication and lifetime antipsychotics

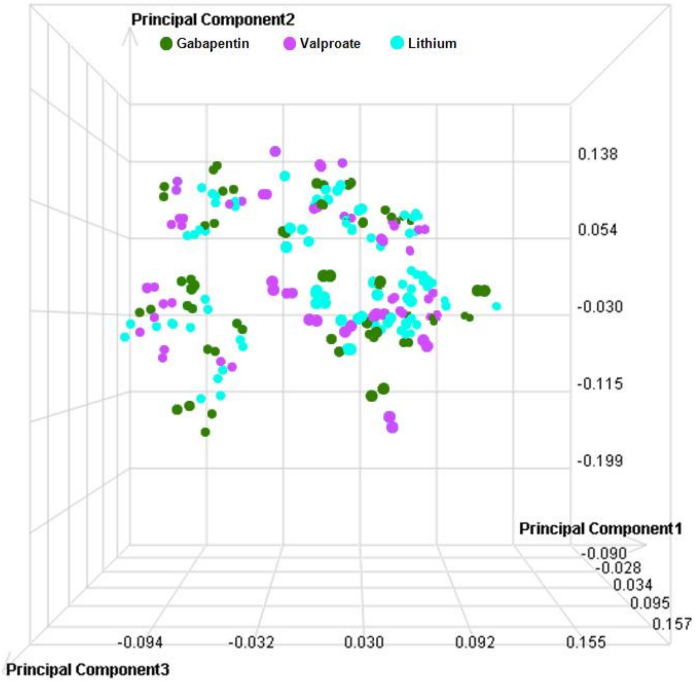

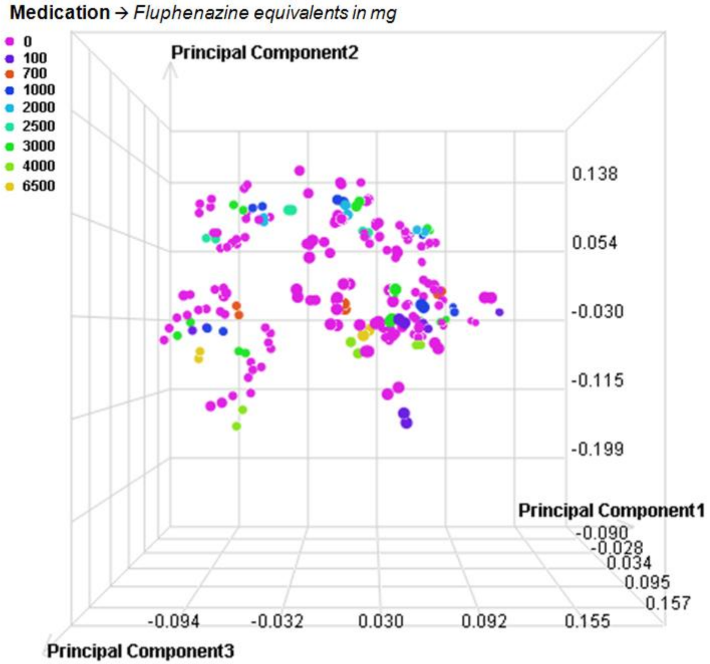

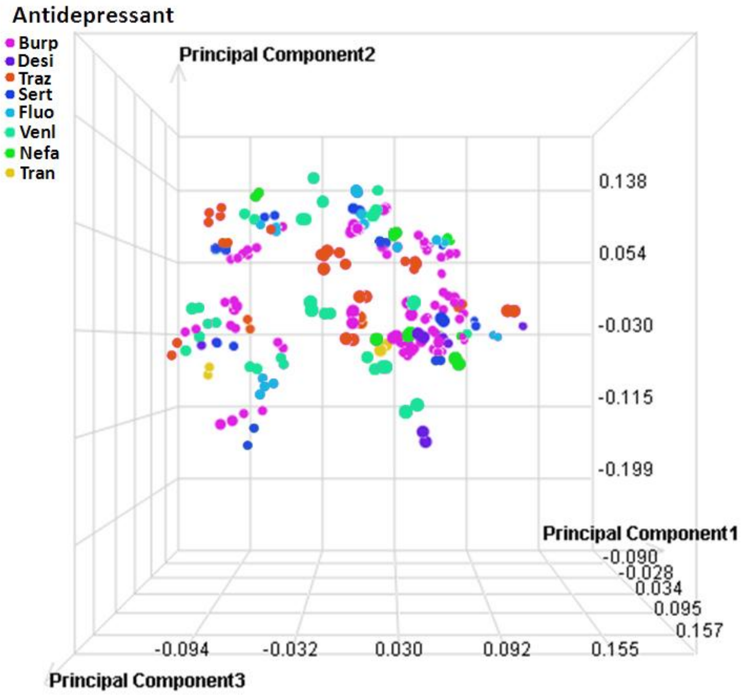

Supplement: Supplementary file 1 — Supplementary material 1 (PDF 426 kb) [file 406_2012_301_MOESM1_ESM.pdf]
